# Supplementary material for: Comorbid Chronic Pain and Depression: Shared Risk Factors and Differential Antidepressant Effectiveness
Source: Front Psychiatry. 2021 Apr 12;12:643609. doi: 10.3389/fpsyt.2021.643609 (PMC8072020; doi:10.3389/fpsyt.2021.643609)
Supplement: Supplementary file 1 [file Data_Sheet_1.docx]

**SUPPLEMENTARY FIGURES**

**
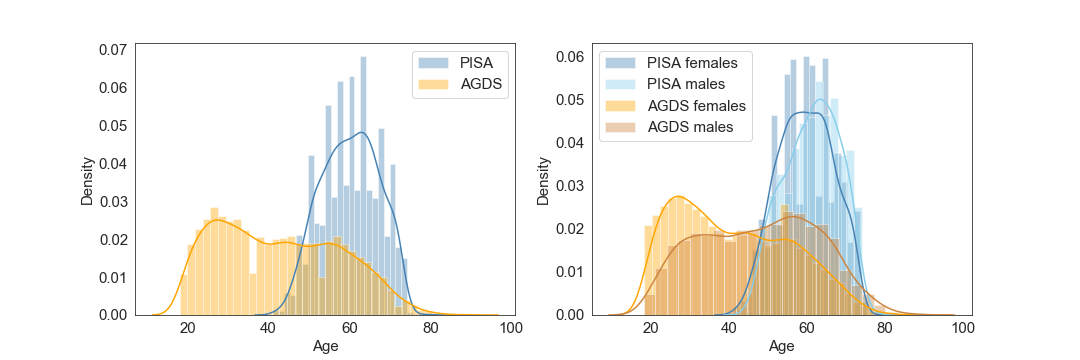
**

**Supplementary Figure 1. Age and sex distributions in AGDS and PISA cohorts**

Histograms with kernel density plots showing **(left)** the distribution of ages for AGDS and PISA cohorts and **(right)** after stratification by sex. Note that the PISA cohort is on average older at the time the participants responded to the questionnaire, but have lower rates of chronic pain on average than the AGDS (depression-enriched) cohort.


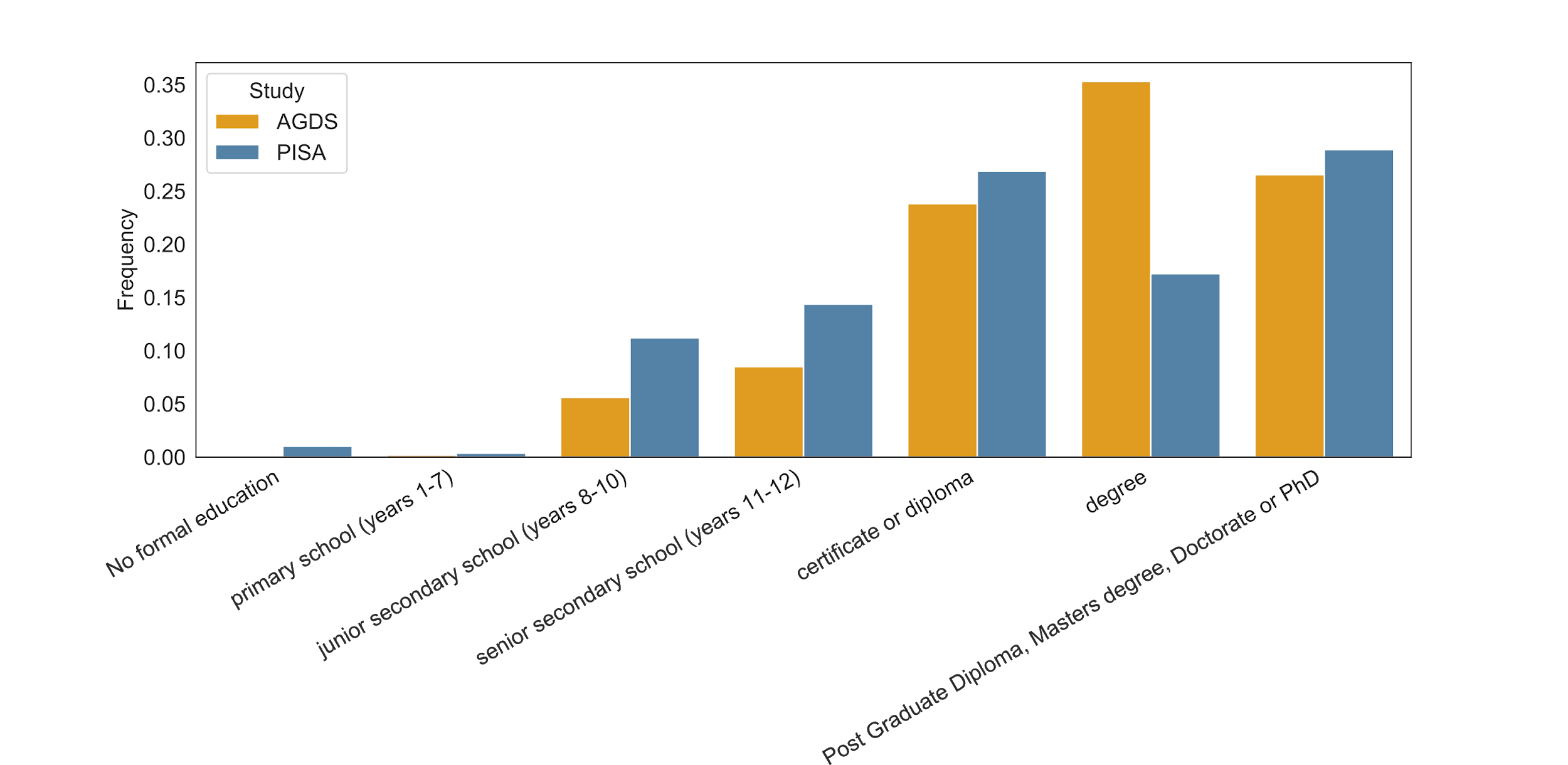


**Supplementary Figure 2. Significant difference in educational attainment between AGDS and PISA cohorts**

Barplots showing the frequency of educational attainment for the AGDS and PISA cohorts. The difference is statistically significant (χ2 test p<E-100).

**
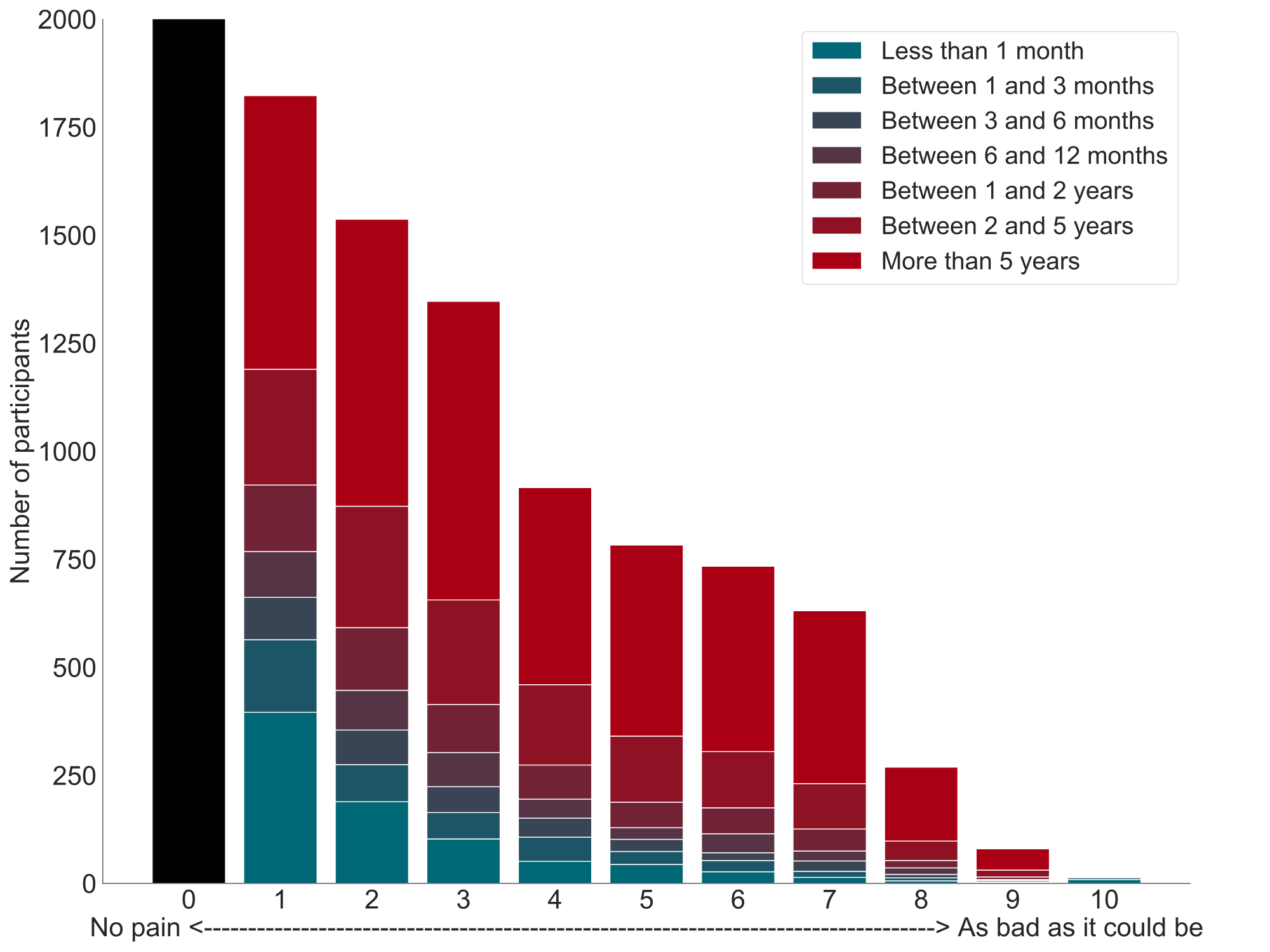
**

**Supplementary Figure 3. Positive association between pain intensity and pain duration in AGDS cohort**

Bar plots illustrate average pain intensity (x-axis) stratified by pain duration, with a strong positive relationship found between these measures (p=5.5e-106 linear regression; AGDS data only).

**
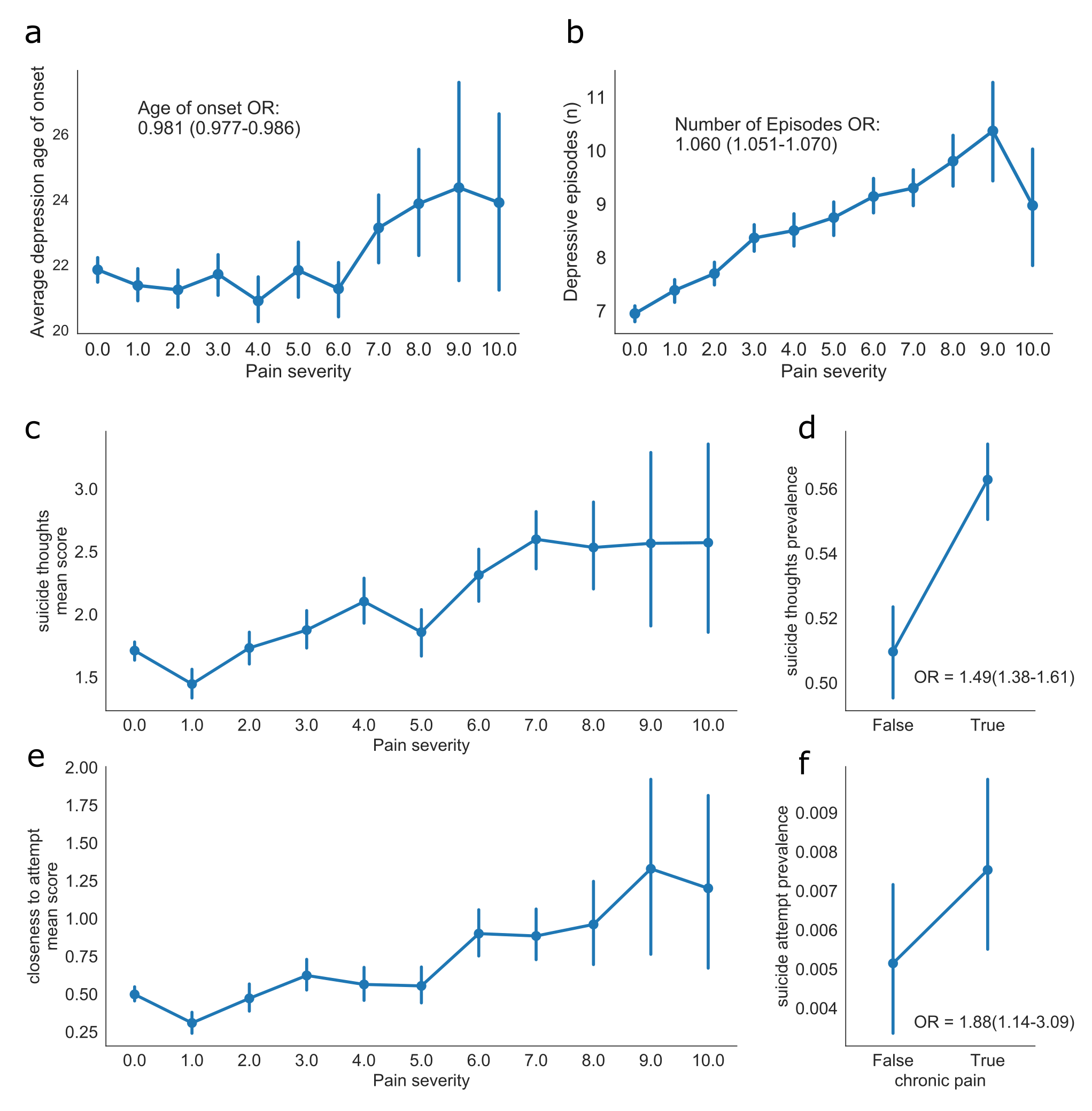
**

**Supplementary Figure 4. Positive association between chronic pain severity (intensity), depression (number of episodes) and suicidality in AGDS cohort**

Point plots comparing chronic pain severity with: **(a)** average age of onset; **(b)** average number of depressive episodes; **(c)** average suicide thoughts score; or **(e)** suicide attempt score. Also shown are differences in the prevalence of **(d)** recent suicide thoughts and **(f)** recent suicide attempts. Error bars denote 95% confidence intervals (AGDS data only).

**
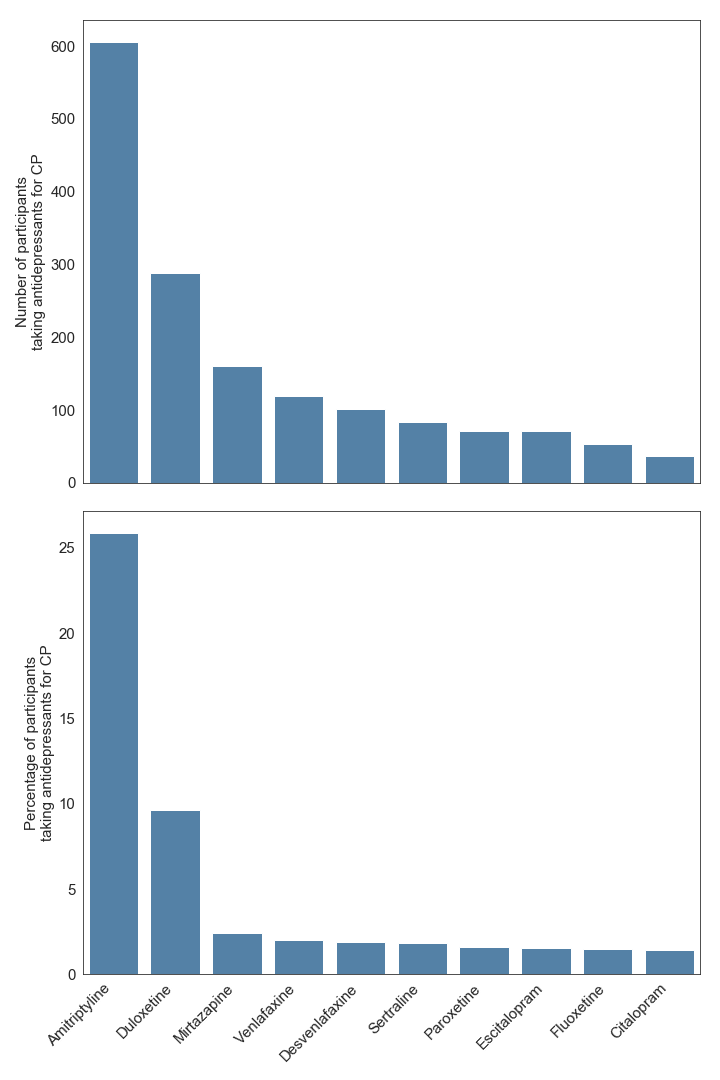
**

**Supplementary Figure 5. Antidepressant intake by chronic pain participants**

Barplots showing the percentage (number) of participants with comorbid chronic pain (CP) and depression taking ten commonly used antidepressants in Australia (AGDS data only).


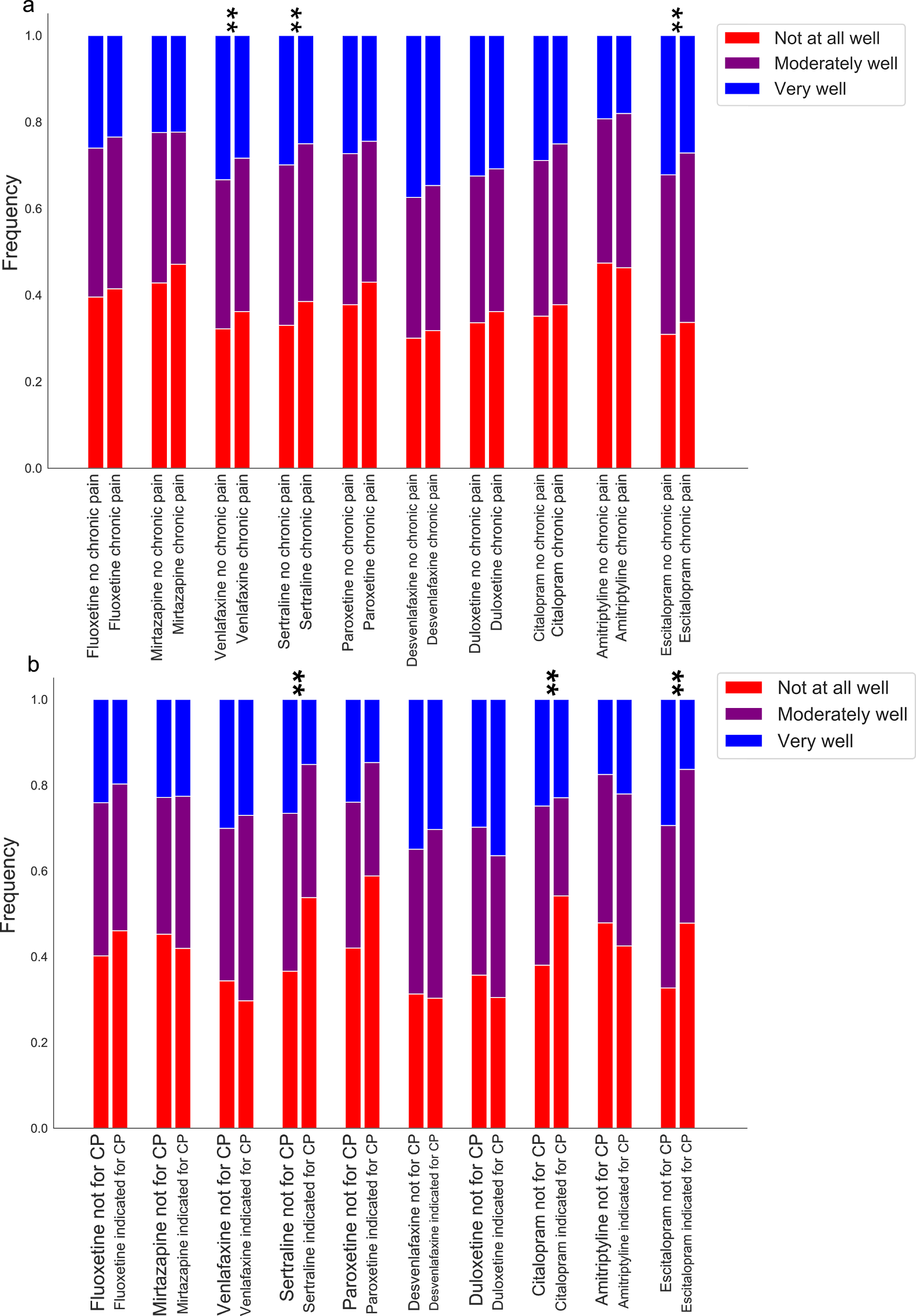


**Supplementary Figure 6. Association between antidepressant response, participants with chronic pain and antidepressant prescription for chronic pain**

Bar plots depicting the average antidepressant response score across antidepressants stratified by participants reporting chronic pain (CP), regardless of: **(a)** the clinical indication for the antidepressant: or **(b)** whether the antidepressant was prescribed for chronic pain. Further details including p-values from a cumulative link logistic regression are in **Supplementary Tables 4 and 5** (AGDS data only).

**SUPPLEMENTARY TABLES**

| **Supplementary Table 1. Effect of demographic variables, depression and cohort on chronic pain** | | |
| --- | --- | --- |
|  | OR (95%CI) | p-value |
| Cohort (AGDS) | 1.31 (0.96–1.77) | 0.086 |
| Sex (female) | 1.16 (1.07–1.26) | 0.0003 |
| Depression* | 1.95 (1.42–2.66) | 2.80E-05 |
| Education | 0.887 (0.86–0.91) | 1.00E-14 |
| Age | 1.02 (1.02–1.03) | 6.90E-74 |
| These results are from a multivariate logistic regression on chronic pain. Fully adjusted ORs are shown. Cohort represents a categorical variable of whether participants were from the AGDS cohort compared to the PISA cohort. *The effect of self-reported depression available on PISA and AGDS (all cases). Note the lack of association with cohort after adjustment for sex, age, education and depression. | | |

| **Supplementary Table 2. Other psychiatric comorbidities with chronic pain** | | |
| --- | --- | --- |
| **Diagnosis** | **OR (95% CI)** | **p-value** |
| Bipolar disorder | 0.947 (0.82–1.09) | 0.4570 |
| Premenstrual dysphoria | 1.19 (0.90–1.57) | 0.2355 |
| Schizophrenia | 1.58 (0.97–2.58) | 0.0675 |
| Anorexia nervosa | 0.788 (0.62–0.99) | 0.0443 |
| Bulimia | 1.17 (0.92–1.49) | 0.2003 |
| ADD/ADHD | 0.826 (0.68–1.01) | 0.0602 |
| Autism/Asperger | 1.14 (0.82–1.60) | 0.4294 |
| Tourette's | 1.12 (0.36–3.49) | 0.8502 |
| Generalized anxiety | 0.999 (0.92–1.09) | 0.9889 |
| Panic disorder | 1.02 (0.88–1.18) | 0.8050 |
| Obsessive-compulsive disorder | 0.945 (0.79–1.13) | 0.5344 |
| Hoarding disorder | 1.29 (0.72–2.32) | 0.3870 |
| PTSD | 0.987 (0.88–1.11) | 0.8335 |
| Phobia | 1.03 (0.82–1.29) | 0.7949 |
| Seasonal disorder | 0.885 (0.70–1.12) | 0.3179 |
| Social anxiety | 1.19 (1.04–1.36) | 0.0096 |
| Agoraphobia | 0.828 (0.63 1.09) | 0.1724 |
| Personality disorder | 1.11 (0.93–1.32) | 0.2440 |
| Substance use | 0.984 (0.79–1.22) | 0.8814 |
| Multivariate logistic regression assessing chronic pain comorbidities.  Multiple testing corrected significance threshold: p<0.0026 | | |

| **Supplementary Table 3. Correlations between recent substance use and chronic pain (CP)** | | |
| --- | --- | --- |
| **Substance** | **CP OR (95%CI)** | **p-value** |
| GHB (gamma hydroxybutyrate) | 0.409 (0.14–1.21) | 0.105 |
| Inhalants | 0.723 (0.54–0.98) | 0.035 |
| Cocaine | 0.811 (0.65–1.01) | 0.060 |
| Ecstasy | 0.857 (0.69–1.06) | 0.161 |
| Other party drugs | 0.886 (0.54–1.46) | 0.632 |
| Stimulants | 0.986 (0.91–1.07) | 0.744 |
| Alcohol | 0.997 (0.99–1.00) | 3.17E-04 |
| Ketamine | 1.02 (0.71–1.45) | 0.933 |
| Hallucinogens | 1.03 (0.76–1.40) | 0.850 |
| E-cigarettes | 1.03 (0.95–1.12) | 0.421 |
| Amphetamines | 1.05 (0.86–1.28) | 0.640 |
| Sedatives or sleeping pills | 1.05 (0.99–1.11) | 0.080 |
| Tobacco | 1.05 (1.02–1.09) | 0.002 |
| Cannabis | 1.07 (1.01–1.13) | 0.016 |
| Painkillers or analgesics* | 1.29 (1.24–1.35) | 1.00E-29 |
| Opioids* | 1.31 (1.06–1.62) | 0.012 |
| Alcohol use was measured as the number of days drinking three or more standard drinks in the past three months. All other variables were measured on a scale ranging from never to daily or almost daily for the past three months.*Non-medical use of opioids & over-the-counter or other painkillers/analgesics | | |

| **Supplementary Table 4. Percentage of chronic pain participants with an antidepressant indication for chronic pain or for depression** | | |
| --- | --- | --- |
| **Antidepressant** | **Chronic pain indication %** | **Depression indication %** |
| Sertraline | 2.83 | 97.29 |
| Escitalopram | 2.62 | 96.45 |
| Venlafaxine | 3.28 | 96.97 |
| Amitriptyline | 31.05 | 61.60 |
| Mirtazapine | 4.37 | 89.78 |
| Desvenlafaxine | 3.31 | 96.96 |
| Citalopram | 2.18 | 95.45 |
| Fluoxetine | 2.46 | 95.35 |
| Duloxetine | 15.32 | 94.95 |
| Paroxetine | 2.11 | 92.67 |

| **Supplementary Table 5. Functional benefits associated with antidepressant use in participants with chronic pain** | | |
| --- | --- | --- |
| **Self-reported benefit** | **OR (95% C.I.)** | **p-value** |
| **Relief of depressive symptoms** | 0.78 (0.71–0.87) | 1.5E-06 |
| **Return of normal emotions** | 0.80 (0.74–0.86) | 2.6E-08 |
| **Getting back to normal daily activities** | 0.82 (0.76–0.89) | 9.2E-07 |
| **Improved relationships with those I am close to** | 0.90 (0.83–0.97) | 0.01 |
| **Restored control over my mood and actions** | 0.90 (0.83–0.98) | 0.01 |
| **Relief of other key symptoms** | 0.94 (0.87–1.02) | 0.15 |
| **Reduction in suicidal thinking or actions** | 1.09 (1.01–1.18) | 0.03 |

| **Supplementary Table 6. Effect of chronic pain on antidepressant response** | | | | |
| --- | --- | --- | --- | --- |
| **Antidepressant** | **N** | **beta** | **S.E.** | **p-value** |
| Sertraline* | 5,189 | -0.29132 | 0.053 | 4.42E-08 |
| Escitalopram* | 3,877 | -0.28702 | 0.061 | 2.96E-06 |
| Venlafaxine* | 3,540 | -0.25168 | 0.065 | 1.13E-04 |
| Amitriptyline | 1,293 | -0.06086 | 0.134 | 0.650 |
| Mirtazapine | 1,718 | -0.16407 | 9.48E-02 | 0.083 |
| Desvenlafaxine | 2,262 | -0.14898 | 0.080 | 0.063 |
| Citalopram | 2,236 | -0.19203 | 0.082 | 0.019 |
| Fluoxetine | 3,178 | -0.1739 | 0.069 | 0.012 |
| Duloxetine | 1,776 | -0.18966 | 0.093 | 0.042 |
| Paroxetine | 1,361 | -0.30056 | 0.107 | 0.005 |
| *p<0.05 after Bonferroni multiple-testing correction for 10 variables/antidepressants | | | | |

| **Supplementary Table 7. Effect of chronic pain indication on antidepressant treatment response** | | | | |
| --- | --- | --- | --- | --- |
| **Antidepressant** | **N** | **beta** | **S.E.** | **p-value** |
| Sertraline* | 5,189 | -0.80599 | 0.207 | 1.00E-04 |
| Escitalopram* | 3,877 | -0.79848 | 0.253 | 0.002 |
| Venlafaxine | 3,540 | 0.143459 | 0.214 | 0.503 |
| Amitriptyline | 1,293 | 0.220429 | 0.119 | 0.064 |
| Mirtazapine | 1,718 | 0.003402 | 0.271 | 0.990 |
| Desvenlafaxine | 2,262 | -0.39922 | 0.270 | 0.140 |
| Citalopram* | 2,236 | -1.14947 | 0.379 | 0.002 |
| Fluoxetine | 3,178 | -0.40595 | 0.268 | 0.130 |
| Duloxetine | 1,776 | 0.2071 | 0.148 | 0.161 |
| Paroxetine | 1,361 | -0.50542 | 0.503 | 0.315 |
| *p<0.05 after Bonferroni multiple-testing correction for 10 variables/antidepressants | | | | |
